# Supplementary material for: Association of part-time clinical work with well-being and mental health in General Internal Medicine: A survey among Swiss hospitalists
Source: PLoS One. 2023 Sep 28;18(9):e0290407. doi: 10.1371/journal.pone.0290407 (PMC10538797; doi:10.1371/journal.pone.0290407)
Supplement: S1 Table — Participants were able to select multiple reasons. *Other reasons listed for part-time clinical employment: administrative work, university duties. (DOCX) [file pone.0290407.s001.docx]

**S1 Table. Reasons for part-time clinical work among hospitalists working part-time and among those working full-time but wishing to reduce to part-time clinical work**

| **Reason for part-time clinical work** | **Full-time employment with wish to reduce to part-time work**  **(n=41)** | **Part-time clinical employment**  **(n=67)** |
| --- | --- | --- |
| Family duties, n (%) | 26 (63%) | 42 (63%) |
| More personal time, n (%) | 37 (90%) | 21 (31%) |
| Other clinical work outside of the hospital, n (%) | 2 (5%) | 3 (4%) |
| Protected research time, n (%) | 7 (17%) | 7 (10%) |
| Protected time for personal education, n (%) | 13 (32%) | 12 (18%) |
| Protected time for teaching, n (%) | 4 (10%) | 7 (10%) |
| Health reasons, n (%) | 4 (10%) | 6 (9%) |
| Other reasons, n (%)* | 1 (2%) | 3 (4%) |

Participants were able to select multiple reasons.

*Other reasons listed for part-time clinical employment: administrative work, university duties
