# Supplementary material for: Association of part-time clinical work with well-being and mental health in General Internal Medicine: A survey among Swiss hospitalists
Source: PLoS One. 2023 Sep 28;18(9):e0290407. doi: 10.1371/journal.pone.0290407 (PMC10538797; doi:10.1371/journal.pone.0290407)
Supplement: S2 Table — Abbreviations: CI: confidence interval; ePWBI, extended Physician Well-Being Index; IQR, interquartile range; OR, odds ratio; SF-8: Short-form Health Survey. Results were adjusted for the propensity of working part-time, as well as age, sex, parenthood, relationship status, academic ambition, reduced work capacity due to health reasons, time since transition to hospitalist, and number of patients to care for * the ePWBI consists of 9 questions and ranges from -2 to 9, with lower scores denoting better well-being. A score of ≥3 points was defined as poor wellbeing.§ measured by a positive answer to at least one of the two first questions of the ePWBI. ♦ rated on a 5-point Likert scale. † measured by a linear analogue scale with a response range from 0 (as bad as can be) to 10 (as good as it can be). ‡ measured by question 9 of the Epwbi. + 8 items with 5- and 6-point Likert-type scales. Each item generates a norm-based T-score ranging from 0 to 100 with higher scores indicating better health, calibrated to a mean score of 50 in the general U.S. population. The Physical and Mental Component Summary is calculated as the weighted sum of the 8 sub-scale scores and normalised to the U.S. general population. ¶ assessed using the 7-item Stanford Sleepiness Scale, 1 being fully alert and 7 imminent sleep onset. # measured using the 2 first items of the 9-item Patient Health Questionnaire (PHQ-2). (DOCX) [file pone.0290407.s002.docx]

**S2 Table.** **Sensitivity analysis considering part-time clinicians working whole weeks (n= 22) as full-time, adjusted for quintiles of the propensity scores.**

|  | **Full-time clinical employment**  **(n=79)** | **Part-time clinical employment**  **(n=45)** | **p-value** |
| --- | --- | --- | --- |
| ePWBI score, mean (95% CI)* | 2.05 (1.06 – 3.03) | 0.9 (-0.42 – 2.21) | 0.09 |
| Poor well-being (ePWBI ≥3), OR (95% CI)* | Ref. | 0.22 (0.06 – 0.88) | **0.032** |
| Symptoms of burnout in the last month, OR (95% CI) § | Ref. | 0.37 (0.12 – 1.19) | 0.10 |
| Job satisfaction, mean (95% CI) ♦ | 3.83 (3.46 – 4.21) | 4.07 (3.6 – 4.54) | 0.31 |
| Quality of life, mean (95% CI) † | 6.4 (5.57 – 7.23) | 6.91 (5.81 – 8.01) | 0.36 |
| Work-life balance, mean (95% CI) ‡ | 0.52 (0.17 – 0.87) | 0.07 (-0.38 – 0.52) | 0.05 |
| Physical Component Summary, mean (95% CI) ^+^ | 52.4 (49.2 – 55.6) | 51.2 (46.9 – 55.4) | 0.57 |
| Mental Component Summary, mean (95% CI) ^+^ | 44.2 (39.7 – 48.7) | 47.7 (41.8 – 53.6) | 0.24 |
| Fatigue, mean (95% CI) ¶ | 1.62 (1.03 – 2.20) | 1.39 (0.64 – 2.14) | 0.55 |
| Depressive symptoms, OR (95% CI) # | Ref. | 0.73 (0.11 – 4.78) | 0.74 |
| Mean daily step count, mean (95% CI) | 8042 (6957 – 9127) | 9687 (7882 – 11492) | 0.07 |

Abbreviations: CI: confidence interval; ePWBI, extended Physician Well-Being Index; IQR, interquartile range; OR, odds ratio; SF-8: Short-form Health Survey

Results were adjusted for the propensity of working part-time, as well as age, sex, parenthood, relationship status, academic ambition, reduced work capacity due to health reasons, time since transition to hospitalist, and number of patients to care for

* the ePWBI consists of 9 questions and ranges from -2 to 9, with lower scores denoting better well-being. A score of ≥3 points was defined as poor wellbeing.

§ measured by a positive answer to at least one of the two first questions of the ePWBI

♦ rated on a 5-point Likert scale

† measured by a linear analogue scale with a response range from 0 (as bad as can be) to 10 (as good as it can be).

‡ measured by question 9 of the ePWBI

^+^ 8 items with 5- and 6-point Likert-type scales. Each item generates a norm-based T-score ranging from 0 to 100 with higher scores indicating better health, calibrated to a mean score of 50 in the general U.S. population. The Physical and Mental Component Summary is calculated as the weighted sum of the 8 sub-scale scores and normalised to the U.S. general population.

¶ assessed using the 7-item Stanford Sleepiness Scale, 1 being fully alert and 7 imminent sleep onset

# measured using the 2 first items of the 9-item Patient Health Questionnaire (PHQ-2).
