# Supplementary material for: Association of part-time clinical work with well-being and mental health in General Internal Medicine: A survey among Swiss hospitalists
Source: PLoS One. 2023 Sep 28;18(9):e0290407. doi: 10.1371/journal.pone.0290407 (PMC10538797; doi:10.1371/journal.pone.0290407)
Supplement: S3 Table — Abbreviations: CI: confidence interval; ePWBI, extended Physician Well-Being Index; IQR, interquartile range; OR, odds ratio; SF-8: Short-form Health Survey. Results were adjusted for the propensity of working part-time, as well as age, sex, parenthood, relationship status, academic ambition, reduced work capacity due to health reasons, time since transition to hospitalist, and number of patients to care for * the ePWBI consists of 9 questions and ranges from -2 to 9, with lower scores denoting better well-being. A score of ≥3 points was defined as poor wellbeing. § measured by a positive answer to at least one of the two first questions of the Epwbi. ♦ rated on a 5-point Likert scale. † measured by a linear analogue scale with a response range from 0 (as bad as can be) to 10 (as good as it can be). ‡ measured by question 9 of the ePWBI. + 8 items with 5- and 6-point Likert-type scales. Each item generates a norm-based T-score ranging from 0 to 100 with higher scores indicating better health, calibrated to a mean score of 50 in the general U.S. population. The Physical and Mental Component Summary is calculated as the weighted sum of the 8 sub-scale scores and normalised to the U.S. general population. ¶ assessed using the 7-item Stanford Sleepiness Scale, 1 being fully alert and 7 imminent sleep onset. # measured using the 2 first items of the 9-item Patient Health Questionnaire (PHQ-2). (DOCX) [file pone.0290407.s003.docx]

**S3 Table**. **Sensitivity analysis considering full-time and part-time employment overall, adjusted for quintiles of the propensity scores.**

|  | **Full-time overall employment**  **(n=71)** | **Part-time overall employment**  **(n=53)** | **p-value** |
| --- | --- | --- | --- |
| ePWBI score, mean (95% CI)* | 1.44 (0.44 – 2.43) | 0.26 (-0.97 – 1.49) | 0.06 |
| Poor well-being (ePWBI ≥3), OR (95% CI)* | Ref. | 0.19 (0.06 – 0.65) | **0.008** |
| Symptoms of burnout in the last month, OR (95% CI) § | Ref. | 0.31 (0.10 – 0.94) | **0.038** |
| Job satisfaction, mean (95% CI) ♦ | 3.90 (3.56 – 4.24) | 4.04 (3.62 – 4.46) | 0.51 |
| Quality of life, mean (95% CI) † | 6.72 (5.88 – 7.55) | 7.39 (6.39 – 8.38) | 0.19 |
| Work-life balance, mean (95% CI) ‡ | 0.31 (-0.05 – 0.68) | -0.22 (-0.66 – 0.22) | **0.018** |
| Physical Component Summary, mean (95% CI) ^+^ | 54.4 (51.3 – 57.6) | 57.1 (53.3 – 60.8) | 0.16 |
| Mental Component Summary, mean (95% CI) ^+^ | 46.4 (41.2 – 50.8) | 50.4 (45.1 – 55.7) | 0.14 |
| Fatigue, mean (95% CI) ¶ | 1.13 (0.57 – 1.69) | 0.39 (-0.29 – 1.06) | **0.030** |
| Depressive symptoms, OR (95% CI) # | Ref. | 0.09 (0.01 – 0.79) | **0.030** |
| Mean daily step count, mean (95% CI) | 8214 (7162 – 9265) | 8595 (7107 – 10084) | 0.60 |

Abbreviations: CI: confidence interval; ePWBI, extended Physician Well-Being Index; IQR, interquartile range; OR, odds ratio; SF-8: Short-form Health Survey

Results were adjusted for the propensity of working part-time, as well as age, sex, parenthood, relationship status, academic ambition, reduced work capacity due to health reasons, time since transition to hospitalist, and number of patients to care for

* the ePWBI consists of 9 questions and ranges from -2 to 9, with lower scores denoting better well-being. A score of ≥3 points was defined as poor wellbeing.

§ measured by a positive answer to at least one of the two first questions of the ePWBI

♦ rated on a 5-point Likert scale

† measured by a linear analogue scale with a response range from 0 (as bad as can be) to 10 (as good as it can be).

‡ measured by question 9 of the ePWBI

^+^ 8 items with 5- and 6-point Likert-type scales. Each item generates a norm-based T-score ranging from 0 to 100 with higher scores indicating better health, calibrated to a mean score of 50 in the general U.S. population. The Physical and Mental Component Summary is calculated as the weighted sum of the 8 sub-scale scores and normalised to the U.S. general population.

¶ assessed using the 7-item Stanford Sleepiness Scale, 1 being fully alert and 7 imminent sleep onset

# measured using the 2 first items of the 9-item Patient Health Questionnaire (PHQ-2).
